# Adrenal Oncocytic Pheochromocytoma: Insights From a Challenging Diagnostic Journey

**DOI:** 10.7759/cureus.67058

**Published:** 2024-08-17

**Authors:** Charusheela Gore, Dipti Singh, Sushama Gurwale, Arpana Dharwadkar

**Affiliations:** 1 Pathology, Dr. D. Y. Patil Medical College, Hospital and Research Centre, Dr. D. Y. Patil Vidyapeeth (Deemed to Be University), Pune, IND

**Keywords:** adrenocortical oncocytomas, oncocytic neoplasm, adrenal oncocytoma, oncocytic pheochromocytoma, oncocytic tumors

## Abstract

Pheochromocytomas are rare adrenal medulla tumors originating from chromaffin cells, accounting for 10% of primary adrenal neoplasms. Oncocytic variants of pheochromocytomas are rare and have been reported in only 150 cases. This report describes the case of a 60-year-old female who arrived with a non-functional adrenal tumor. This case report emphasizes the importance of a comprehensive histological and immunohistochemical study for diagnosing this rare diagnostic entity and its potential diagnostic pitfalls.

## Introduction

Pheochromocytomas are rare adrenal medulla tumors that arise from chromaffin cells. These tumors originate from the embryonic neural crest and approximately 10% of primary adrenal neoplasms are pheochromocytomas [1]. Specific anatomic areas with histologic and ultrastructural features characteristic of their catecholamine-secreting tumor type include big polygonal cells, thick granular eosinophilic cytoplasm, and numerous mitochondria packed firmly into the cell. Since the first instance was identified in 1997 and given the name oncocytic pheochromocytoma, there have been few occurrences of the condition recorded [2].

Oncocytic tumors have been documented in several organs, including the kidney, thyroid, parathyroid, salivary glands, and pituitary [3]. Their histology demonstrates nesting, alveolar, and trabecular patterns [4]. Neuroendocrine markers such as chromogranin and synaptophysin are detected positively by immunohistochemistry [4,5].

Adrenal oncocytic tumors are extremely uncommon and only approximately 150 cases have been reported [6]. Of these, six cases were oncocytic tumors of the adrenal medulla: five pheochromocytomas and one adrenal medullary oncocytoma [7]. Since oncocytic forms of adrenal pheochromocytomas are exceedingly uncommon [8], they have not been included in recent research on adrenal oncocytic neoplasms [9].

## Case presentation

**Figure 1:**
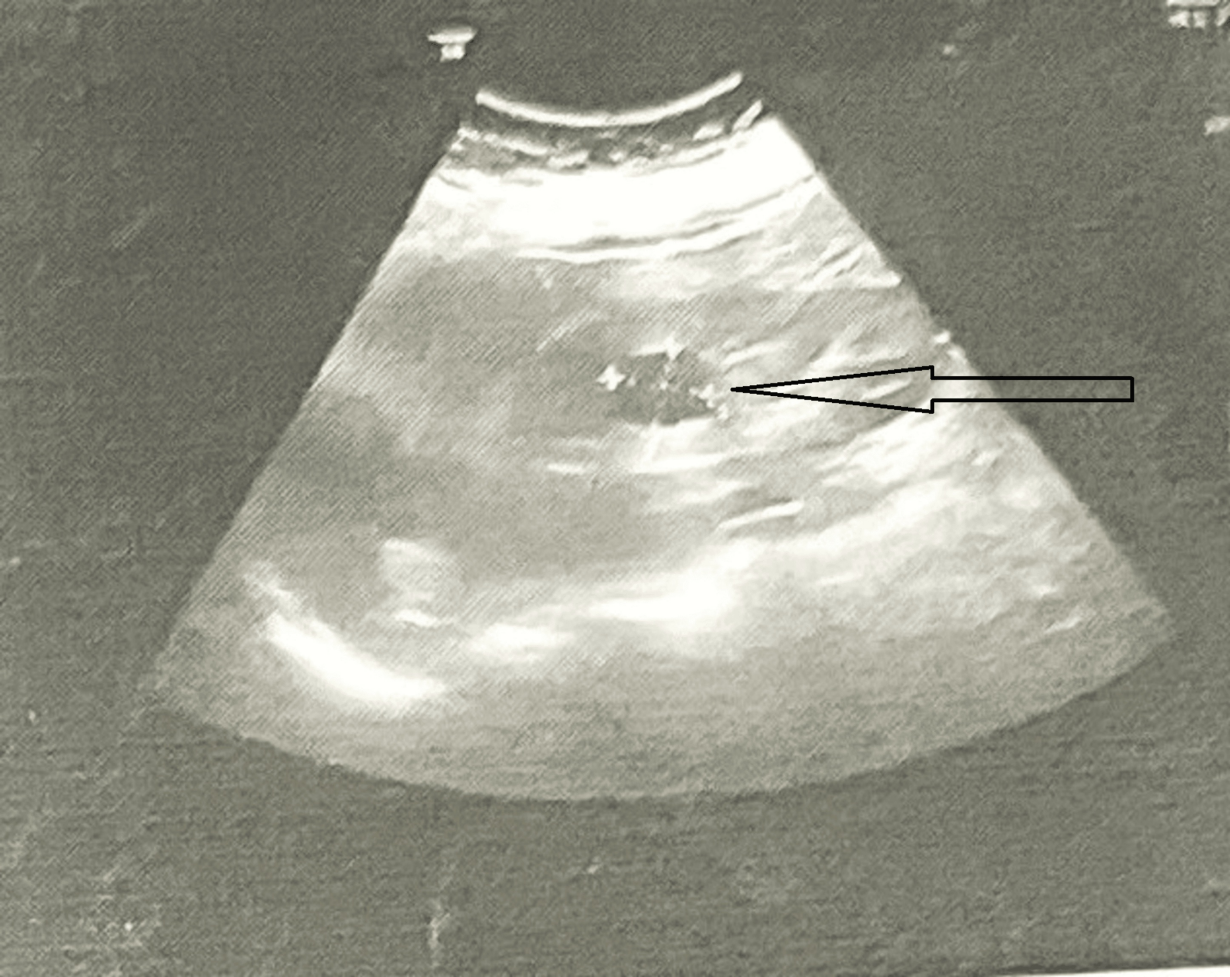
Ultrasound of the abdomen showing a left renal cystic lesion A cystic lesion measuring 23 x 23 mm without internal septations/ echoes was noted in the mid-pole region, suggesting a simple corticomedullary cyst (arrow).

A 60-year-old female experienced left-side flank discomfort, weight loss, and intermittent burning during micturition for the past three months. She had a right-side nephrectomy 10 years ago for a non-functioning kidney with staghorn calculus and mild hypertension. An ultrasonographic examination revealed a cystic lesion along the medial side of the left kidney with internal echoes, raising suspicion of either a renal parapelvic cyst or an adrenal cyst (Figure 1)*.*

**Figure 2:**
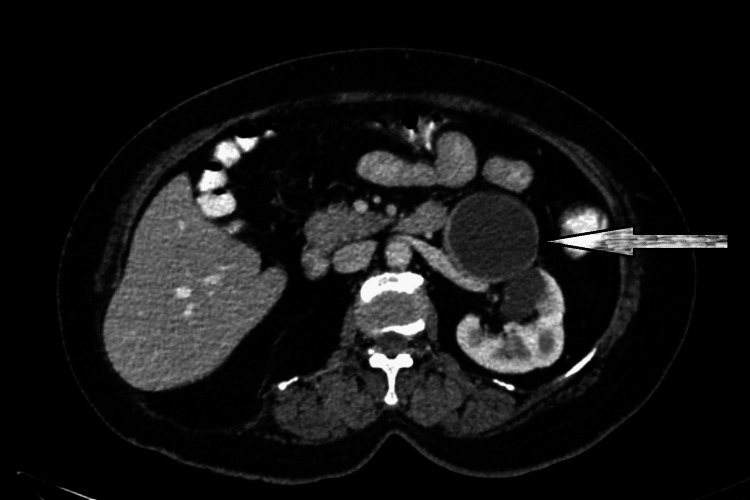
CT with contrast, axial section abdomen showing left side pararenal and renal space occupying lesion (SOL) A CT scan showed a peripherally enhanced hypoattenuating large cystic left adrenal mass in the retroperitoneum and along the inferolateral aspect of the left adrenal gland, seen abutting the pancreatic tail (arrow). Metastatic deposits or masses were not found.

A CT contrast revealed a 71 x 61 x 56 mm peripherally enhanced hypoattenuating cystic left adrenal mass in the retroperitoneum near the tail of the pancreas (Figure 2). The right kidney was not visualized, which was consistent with a history of nephrectomy.

**Figure 3:**
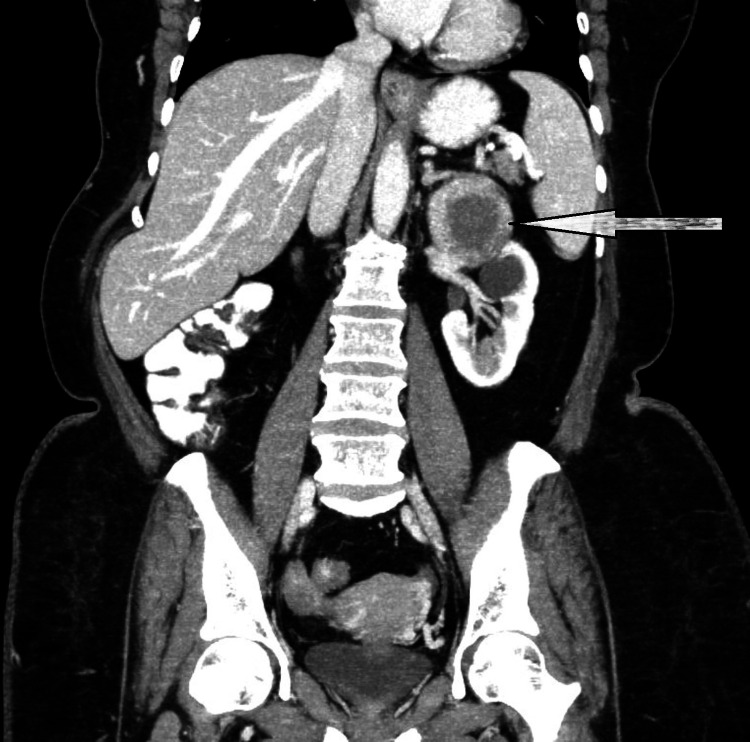
Coronal CT of abdomen and pelvis A large well-defined hypoattenuating, non-enhancing cystic lesion of size 71 x 61 x 56 mm, surrounded by enhancing adrenal tissue was noted arising from the inferolateral aspect of the left adrenal gland, seen abutting the tail of the pancreas and the small bowel loop (arrow). It showed no solid components/septations/calcifications.

Left side simple renal cortical cyst with parapelvic cyst was identified. No other masses or metastatic deposits were found (Figure 3). The patient was diagnosed with hypertension (blood pressure near 140-160/80-90 mm Hg) for four years and has been effectively managed with medication. The pre-operative biochemical evaluation showed normal adrenalin and nor-adrenalin levels in urine and plasma (Table 1). With a clinical diagnosis of a non-functional adrenal cystic lesion, surgical excision was performed.

**Table 1:** Biochemical evaluation for the present case Urine and plasma levels in case of biochemical evaluation for adrenal tumors. Remember that these values can vary slightly based on the laboratory reference ranges and the specific assay used.

Serial No.	Test name	Observed values	Reference values
1	Vanillylmandelic acid (VMA) in urine	2.8 mg/24 hour	2-7 mg/24 hours
2	Adrenaline (epinephrine) in urine	3 µg/24 hour	0-20 µg/24 hours
3	Noradrenaline (norepinephrine) in urine	20 µg/24 hour	15-80 µg/24 hours
4	Metadrenalin in plasma	0.42 nmol/L	<0.5 nmol/L
5	Normetadrenalin in plasma	0.36 nmol/L	<0.9 nmol/L
6	Aldosterone in plasma	85 pmol/L	55-440 pmol/L
7	Cortisol in plasma (morning)	9 µg/dL	5-23 µg/dL
8	Adrenocorticotropic hormone (ACTH) in plasma	5.3 pmol/L	2.2-13.2 pmol/L
9	Renin in plasma	0.21 ng/mL/hour	0.2-2.3 ng/mL/hour

**Figure 4:**
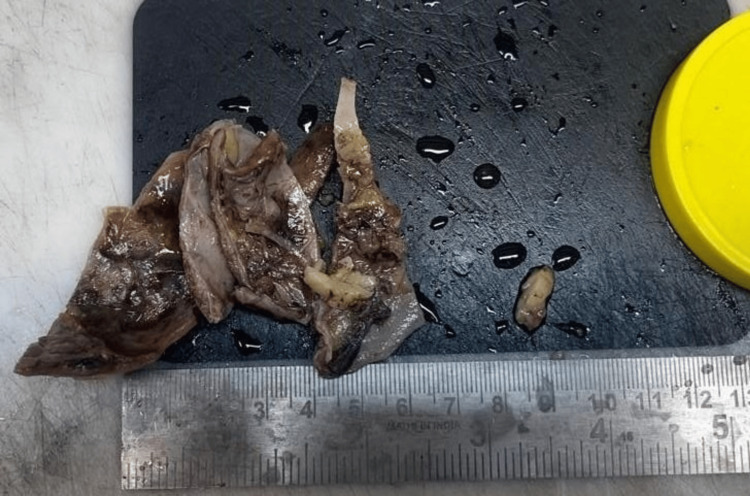
Specimen (cystic adrenal lesion) received for histopathology Gross section showing greyish white external surface covered with fibro-fatty tissue having few hemorrhagic areas and measuring 75 x 60 x 30 mm.

A gross examination of the received specimen revealed a well-circumscribed, cystic, solid adrenal mass measuring 75 x 60 x 30 mm and weighing 15 grams. The external surface was grayish-white and covered with yellow fibrofatty tissue (Figure 4). The cut surface was cystic with a shiny thinned-out wall; a few thickened areas in the wall of the cyst were noted, which appeared yellow; and a few hemorrhagic regions were also noted.

**Figure 5:**
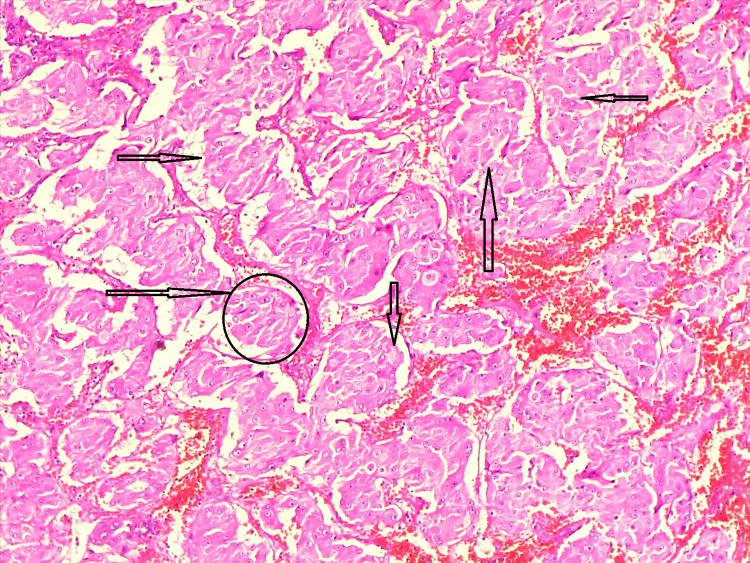
Microscopic examination of the adrenal cystic tumor (hematoxylin & eosin stain, 10x) The polygonal cells had a lot of granular eosinophilic cytoplasm and partly pleomorphic nuclei with nucleoli. Arrows are placed in the surrounding septa around a bunch of cells. Necrosis or an increase in mitotic activity was not visible. There was a zellballen pattern (circle), which is indicative of pheochromocytoma.

Histopathological findings showed adrenal tissue and a cystic tumor. The wall of which comprised of nests separated by thin fibrovascular stroma (Figure 5). The cells were polygonal and showed mildly pleomorphic nuclei with small nucleoli and abundant granular eosinophilic cytoplasm. There was no evidence of necrosis or increase in mitotic activity (Figure 5). In this case, adrenal cortical adenoma, adrenal cortical cancer, medullary oncocytoma, adrenal cortical cyst, pheochromocytoma, and oncocytic pheochromocytoma were the differential diagnoses for adrenal oncocytic pheochromocytoma. A provisional diagnosis of pheochromocytoma/oncocytic pheochromocytoma was offered by correlating clinicopathological and immunohistochemistry findings.

**Figure 6:**
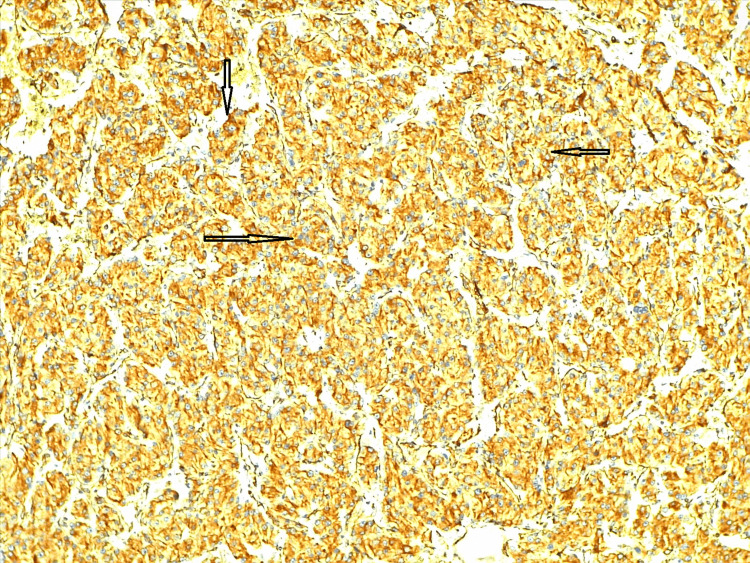
Immunohistochemistry reveals vimentin-positive tumor cells Immunohistochemistry staining of oncocytic pheochromocytoma showing positive vimentin expression (arrows). Vimentin, a mesenchymal marker, is detected in the tumor cells (brown staining). Areas with denser brown coloration indicate higher vimentin expression.

**Figure 7:**
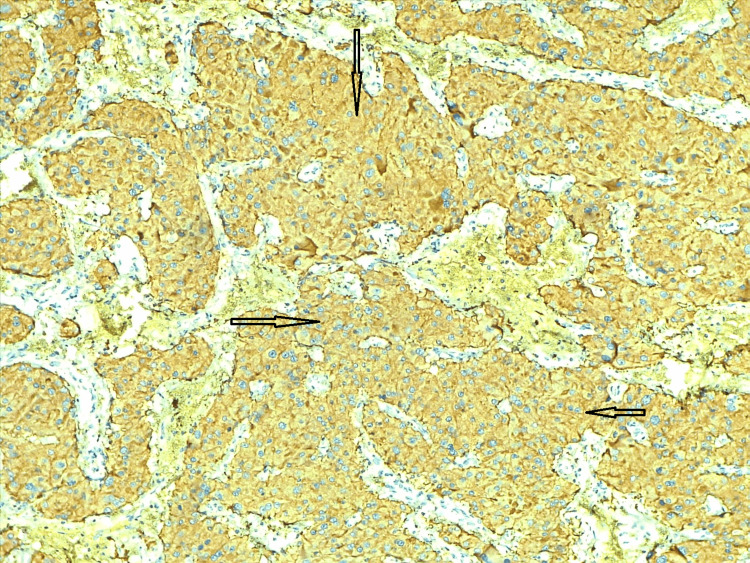
Immunohistochemistry - synaptophysin Immunohistochemistry shows synaptophysin identified the oncocytic component (arrows). Pheochromocytoma and paragangliomas are usually positive for synaptophysin.

**Figure 8:**
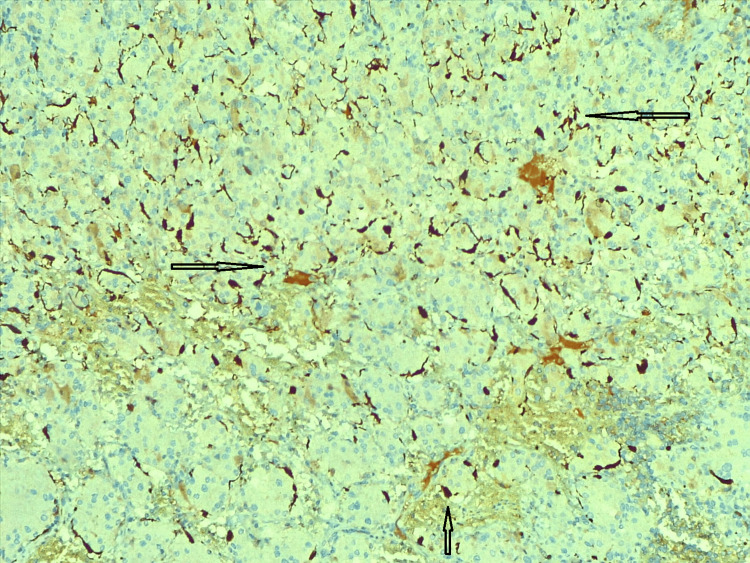
Immunohistochemistry - S100 The oncocytic pheochromocytoma adrenal gland component was identified by immunohistochemistry, with S100 shown in the figure (arrows). Sustentacular cells showed positivity for S100 IHC. IHC: immunohistochemistry

**Figure 9:**
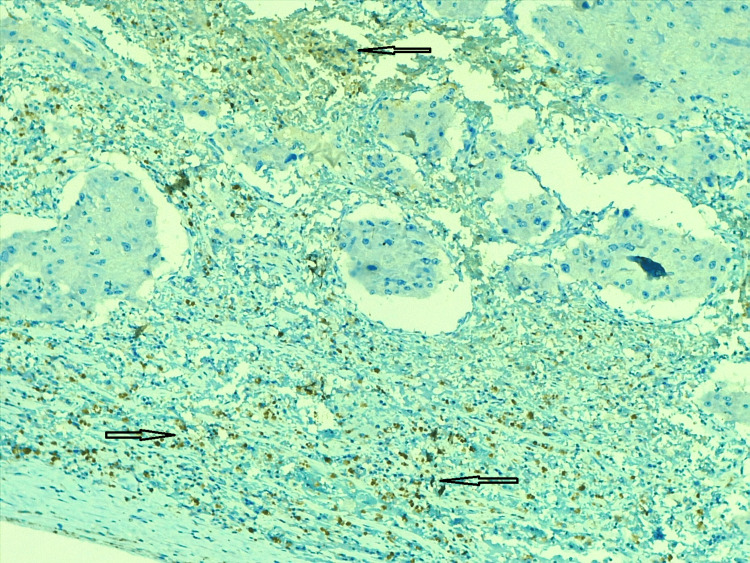
Immunohistochemistry - pan CK Immunohistochemistry revealing pan CK identified the oncocytic pheochromocytoma component (arrows). Some of the cells are showing (weak) positive (brown) for pan CK.

Immunohistochemical markers like vimentin (Figure 6), synaptophysin (Figure 7), S100 (Figure 8), and pan CK (Figure 9) were positive in sustentacular cells. These findings confirmed the diagnosis of a cystic variant of oncocytic pheochromocytoma. There was no incident throughout the recovery time. The patient has not reported any new problems throughout the last five months of routine periodic follow-up. Her abdominal discomfort has lessened, and now she has resumed her normal activities.

## Discussion

Oncocytic pheochromocytoma is clinically nonfunctional and has an unclear cause. Oncocytic change is a degenerative phenomenon where mitochondria accumulate to compensate for oxidative phosphorylation uncoupling, leading to nonfunctional oncocytic tumors [10]. The literature provides six previous reports of oncocytic pheochromocytoma (Table 2) [2,4,5,10-12].

**Table 2:** Reports in the literature on the features of oncocytic pheochromocytoma M: male; F: female; L: left; R: right; G: gram; mm: millimeter

Demographic (author/year/country)	Sex/age (years)	Side	Clinical presentation	Weight	Size	Follow up	Remarks	Histopathological specific features
Wang et al./1997/USA [2]	F/38	L	Acute abdominal pain and hirsutism	275 G	34 mm	Nil	Areas of hemorrhage, cytokeratin+	Fibrovascular stroma surrounding Nest of tumor cells, nuclear atypia
Li and Wenig/2000/USA [4]	F/37	L	Increase abdominal girth	1150 G	170 mm	20 months	Pseudoglandular pattern	Psudoglandular pattern, a large nucleus with prominent nucleolus
Shibamori et al./2013/Japan [5]	F/61	R	Incidental finding	260 G	100 mm	13 months	Alveolar configuration	Alveolar configuration, round nucleus
Kasem and Lam/2014/Australia [10]	M/68	L	Incidental finding	36 G	45 mm	5 years	P53+	Myelolipomatous metaplasia, capsular invasion
Nam et al./2014/USA [11]	M/81	L	Incidental finding	NA	NA	NA	CT guided fine needle aspiration	Sheets of cells, nuclear pleomorphism
Kenneth/2018/Wisconsin [12]	M/54	L	Hypertensive episode	675 G	135 mm	3 months	Necrotic with capsular invasion	Nuclear pleomorphism with nuclear pseudo-inclusions
Present Case/2024/India	F/60	L	Abdominal pain	15G	75 mm	5 months	Hemorrhage with the cyst, nesting pattern	pleomorphic nuclei with nucleoli and abundant granular eosinophilic cytoplasm

In this case, the differential diagnosis among adrenal cortical cyst, pheochromocytoma, and oncocytic pheochromocytoma was crucial. Initially, a diagnosis of cortical cystic pheochromocytoma was proposed; however, immunohistochemistry findings ruled out any cortical components [5]. An immunohistochemical study indicated that this tumor was pheochromocytoma because of the neuroendocrine markers, such as vimentin, synaptophysin, S100, pan CK, and CD-56. Adrenal cortical tumors frequently test positive for SF-1 and inhibin. However, SF-1 is not widely available or used, but inhibin-negative immunohistochemistry can be observed in some adrenocortical tumors [13]. Table 2 summarizes the characteristics of oncocytic pheochromocytoma from the literature.

Documented oncocytic pheochromocytoma patients do not show signs of excessive adrenaline, such as palpitations, flushing, diaphoresis, headaches, or hypertension. In three cases, hypertension was not caused by excess catecholamines. Chemical workups for pheochromocytomas showed no significant increase in catecholamines or their metabolites, indicating that oncocytic pheochromocytomas are "silent" and nonfunctional. These patients had an excellent prognosis and the tumors were clinically nonfunctional, according to the previously published research results (Table 2). Histologically, neither capsular nor lympho-vascular invasion was present, nor was there an increase in mitosis. It was difficult to identify the type of tumor - benign or malignant - until a thorough microscopic examination showed patches of hemorrhage, coagulation necrosis, and fibrotic degradation.

Oncocytic tumors share cytological characteristics such as rich eosinophilic granular cytoplasm and central or eccentric nuclei. The malignancies can be distinguished using immunohistochemical investigations. For instance, pheochromocytomas are specific for chromogranin A and CD56, while an adrenocortical origin would be suggested by positive for calretinin, MART-1, and inhibin A. Synaptophysin levels can be elevated in both pheochromocytomas and adrenal cortical tumors [14].

Pheochromocytoma's aggressive potential is difficult to predict. Two major scoring systems exist Thompson's PASS, based on histological criteria, and Kimura's Japanese scoring system, which includes Ki-67 immunoreactivity and catecholamine types, based on histological criteria and Ki-67 immunoreactivity [15]. PASS score includes histopathological characteristics like large nests, diffuse tumor necrosis, high cellularity, cell monotony, spindling, mitotic figures, extension into adipose tissue, vascular and capsular invasion, profound nuclear pleomorphism, and nuclear hyperchromasia for determining the aggressive nature of the lesion. A score of more than 4/20 is considered malignant [16]. The current case of pheochromocytoma is benign, showing large nests or diffuse growth exceeding 10% resulting in a PASS score of less than four.

## Conclusions

Oncocytic pheochromocytomas are usually non-functional tumors with unique histological and ultrastructural features. They are often found incidentally and are differentiated from other adrenal gland tumors like adrenocortical oncocytomas and metastatic oncocytic tumors. Immunohistochemistry confirms the diagnosis by demonstrating neuroendocrine markers. This case underscores the need for a thorough diagnostic strategy for detecting and managing rare adrenal tumors, including accurate differentiation from other adrenal neoplasms and diligent follow-up.
